# Rapidly Progressive Overlap of Immune Checkpoint Inhibitor-Induced Myositis and Myasthenia Gravis: Diagnostic and Therapeutic Challenges

**DOI:** 10.7759/cureus.102739

**Published:** 2026-01-31

**Authors:** Sofia Sequeira, Catarina Borges, Paulo Castro, Nelson Barros, Michel Mendes

**Affiliations:** 1 Internal Medicine, Hospital de Santo Espírito da Ilha Terceira, Angra do Heroísmo, PRT; 2 Neurology, Unidade Local de Saúde de Trás-os-Montes e Alto Douro, Vila Real, PRT; 3 Oncology, Unidade Local de Saúde de Trás-os-Montes e Alto Douro, Vila Real, PRT; 4 Intensive Care Medicine, Unidade Local de Saúde de Trás-os-Montes e Alto Douro, Vila Real, PRT

**Keywords:** immune-checkpoint inhibitors, immune-related adverse events, myasthenia gravis, myositis, neuromuscular toxicity, overlap syndrome

## Abstract

Immune checkpoint inhibitors (ICIs) are increasingly used across advanced malignancies but may precipitate severe immune-related neuromuscular toxicities, particularly with combination regimens. We report a fulminant case of overlap syndrome involving ICI-induced myositis and myasthenia gravis in a 72-year-old man receiving ipilimumab-nivolumab for hepatocellular carcinoma and recurrent renal cell carcinoma. Shortly after the second cycle, he developed rapidly progressive ophthalmoparesis, bulbar dysfunction, myalgias, and proximal tetraparesis. Laboratory studies revealed marked elevations of creatine kinase, myoglobin, and troponin, and electromyography demonstrated a myopathic pattern with active denervation. Acetylcholine receptor antibodies were positive, raising suspicion for a concomitant ICI-associated myasthenic component within an overlap neuromuscular syndrome. Despite prompt intensive care admission and treatment with high-dose intravenous methylprednisolone and intravenous immunoglobulin, only partial clinical improvement was achieved. The subsequent development of aspiration pneumonia led to acute respiratory and hepatic failure, culminating in death. This case underscores the aggressive clinical trajectory and high mortality of ICI-related neuromuscular overlap syndromes, highlighting the critical need for early recognition, rapid immunosuppression, and multidisciplinary management.

## Introduction

Immune checkpoint inhibitors (ICIs) consist of monoclonal antibodies that target proteins regulating the immune system, aiming to optimize the immune response to cancer cells [1]. These novel medications have transformed the management of several advanced malignancies, and their use has become increasingly widespread. However, by disrupting immune tolerance through sustained T-cell activation and inhibition of regulatory immune checkpoints, ICIs may trigger immune-related adverse events (irAEs) resulting from off-target immune-mediated inflammation affecting multiple organ systems. According to severity, irAEs are classified into five grades: 1: asymptomatic or mild symptoms; 2: moderate symptoms with limitation in instrumental activities of daily living; 3: severe and incapacitating symptoms limiting basic daily activities but not immediately life-threatening; 4: severe and life-threatening events; and 5: death due to irAEs [2].

While cutaneous irAEs are the most prevalent [3], neurological and cardiac toxicities are less frequent but often severe [4]. Neurological irAEs occur in approximately 1%-5% of patients and typically develop within 6-13 weeks after treatment initiation [4]. Among the spectrum of neurotoxicity, neuromuscular irAEs-including acute immune demyelinating polyneuropathy, chronic immune demyelinating polyneuropathy, cranial nerve neuropathies, myasthenic syndromes, and myositis-account for nearly 50% of cases [5].

ICI-induced myositis, the most frequent peripheral nervous system toxicity, is an early-onset, T-cell-mediated necrotizing inflammatory myopathy that often presents within the first weeks of treatment [6,7]. It may manifest as asymptomatic creatine kinase (CK) elevation or as proximal and axial muscle weakness, sometimes preceded by myalgias. In 40%-50% of cases, isolated bulbar or oculomotor involvement may occur. A particularly distinctive and life-threatening feature of ICI toxicity is the occurrence of overlap syndromes involving myositis, myocarditis, and myasthenia gravis, which are associated with markedly increased morbidity and mortality [8-10].

ICI-associated myasthenia gravis occurs in approximately 14% of neurological irAEs and typically presents with abrupt onset, early bulbar and respiratory involvement, and significantly higher fatality rates compared with idiopathic myasthenia gravis. Acetylcholine receptor antibodies may be detected in a subset of patients [8]. We report a fulminant case of ICI-induced necrotizing myositis with overlapping myasthenia gravis following combination ipilimumab-nivolumab therapy.

## Case presentation

A 72-year-old male patient with good baseline functional status (Eastern Cooperative Oncology Group (ECOG) [11] Performance Status 0) was under oncologic follow-up for hepatocellular carcinoma and a late recurrence of renal cell carcinoma, both considered active at the time of ICI initiation. The patient had previously undergone nephrectomy for renal cell carcinoma several years earlier and had remained in remission until the late recurrence. Given the presence of two active malignancies and limited therapeutic options, combined ICI therapy with ipilimumab and nivolumab was initiated in a palliative setting in December 2023.

Symptoms began approximately three days before hospital admission (day −3), with progressive diplopia, dysphagia, myalgias, and proximal limb weakness. The patient was admitted to the hospital on day 0, shortly after receiving the second cycle of ipilimumab-nivolumab in March 2024. Initial laboratory evaluation revealed marked elevations of muscle and cardiac biomarkers, including CK, myoglobin, and troponin T (1.34 ng/mL), consistent with severe skeletal muscle injury and raising concern for myocardial involvement (Table 1). Acetylcholine receptor antibodies were positive, while anti-MuSK antibodies were negative.

**Table 1:** Laboratory findings at presentation

Laboratory parameter	Patient value	Normal reference range	Interpretation
Creatine kinase (CK)	15,480 U/L	<190 U/L	Markedly elevated, consistent with severe myositis
Myoglobin	10,839 ng/mL	<72 ng/mL	Severe muscle breakdown
Troponin T	1.34 ng/mL	<0.05 ng/mL	Suggestive of myocardial involvement
Acetylcholine receptor antibodies	2.76 nmol/L	<0.50 nmol/L	Positive, supporting immune-mediated myasthenia gravis
Anti-MuSK antibodies	0.0054 nmol/L	<0.05 nmol/L	Negative

Neurological examination demonstrated complete ophthalmoplegia, bilateral ptosis, facial palsy, and proximal-predominant tetraparesis. Classical fatigability could not be reliably assessed due to the severity of neurological deficits at presentation, including profound ophthalmoplegia and marked proximal weakness. Although repetitive nerve stimulation was not performed, the combination of early and prominent ocular and bulbar involvement together with acetylcholine receptor antibody positivity supported the suspicion of a myasthenic component within an ICI-related overlap neuromuscular syndrome, rather than isolated myositis.

**Figure 1:**
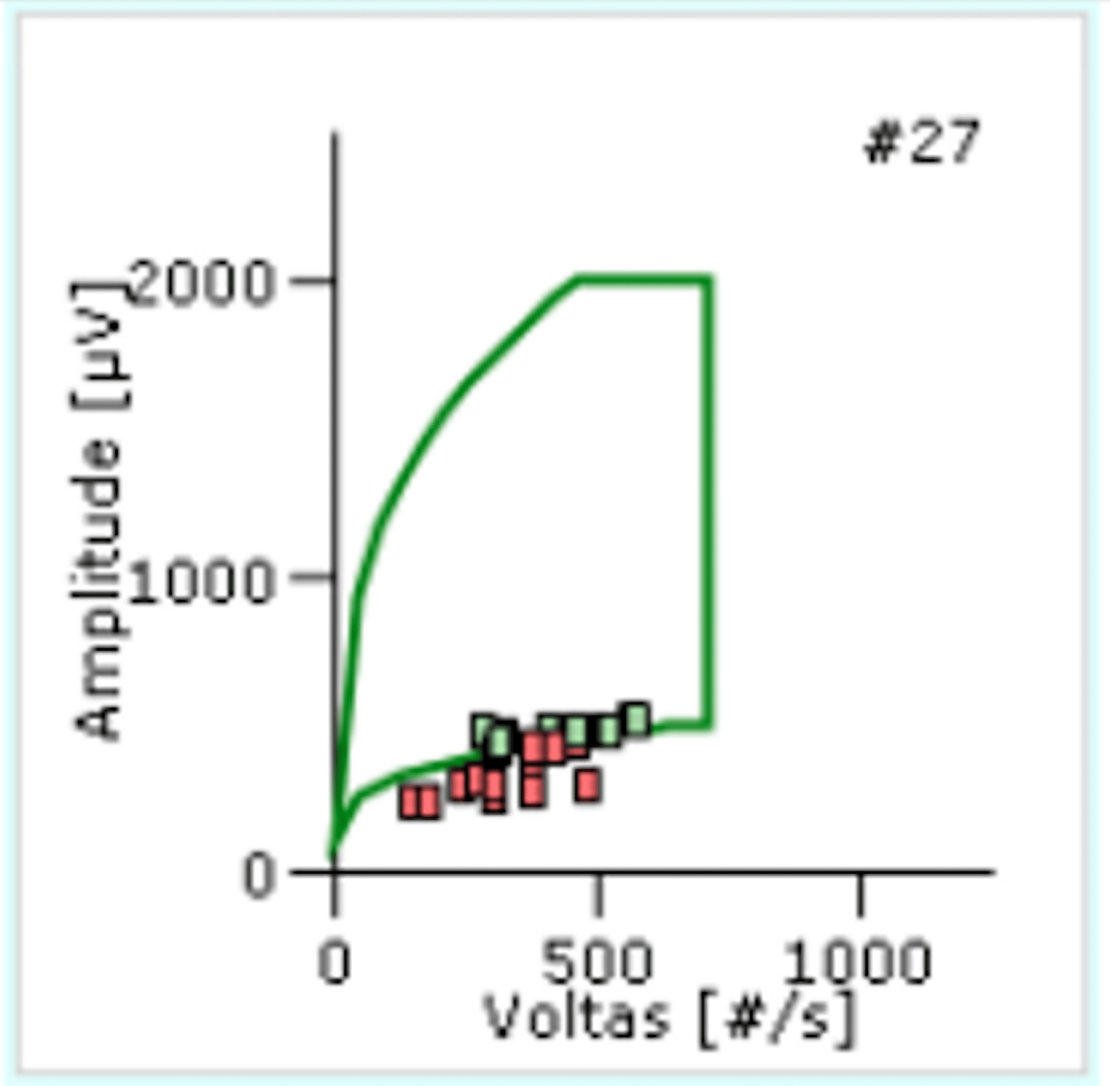
Electromyography findings from the right tibialis anterior muscle. Motor unit action potential cloud plot demonstrates reduced amplitude and short duration with early recruitment, consistent with inflammatory myopathy

Electromyography demonstrated a myopathic pattern with active denervation, consistent with inflammatory myositis (Table 2 and Figure 1). Repetitive nerve stimulation was not performed due to the severity of neuromuscular involvement and rapid clinical deterioration, which limited the feasibility and potential diagnostic yield of the examination.

**Table 2:** Electromyographic findings consistent with inflammatory myopathy On electromyography, we identified low-amplitude potentials with normal duration and excessive polyphasia suggestive of myopathy. The presence of active denervation supports muscle fiber damage, which is consistent with an inflammatory myopathic process. Fibrillation potentials and positive sharp waves are reported as the number of abnormal insertional sites out of 10 examined (e.g., 02/10, 03/10, and 08/10). Complex repetitive discharges are graded semi-quantitatively using a standard electromyography scale (1+ to 4+), and “–” indicates the absence of these discharges. MUAP: motor unit action potential

		Spontaneous activity			Voluntary activity						
Muscle examined	Interpretation	Fibrillation potentials	Positive sharp waves	Complex repetitive discharges	MUAP amplitude	MUAP duration	Polyphasia	Stability	Interference pattern	Recruitment	Comments
Right biceps	Myopathy	02/10	02/10	2+	Reduced	Normal	Increased	Normal	Normal	Reduced	Myopathic pattern
Right extensor digitorum	Myopathy	03/10	03/10	–	Normal	Normal	Increased	Normal	Normal	Reduced	Myopathic pattern
Right tibialis anterior	Myopathy	08/10	08/10	1+	Reduced	Normal	Increased	Normal	Normal	Reduced	Myopathic pattern
Right vastus medialis	Myopathy	08/10	08/10	–	Reduced	Normal	Increased	Normal	Normal	Reduced	Myopathic pattern

Due to rapid clinical deterioration, the patient was transferred to the intensive care unit on day 2 with a diagnosis of grade III ICI-induced myositis with suspected overlap neuromuscular involvement. High-dose intravenous methylprednisolone (1 g/day) was initiated on day 2, and intravenous immunoglobulin at a dose of 0.4 g/kg/day was promptly added from day 3 to day 7 following recognition of severe neuromuscular involvement. During hospitalization, the patient developed type I respiratory failure requiring non-invasive ventilation.

Despite the marked elevation of troponin T suggesting myocardial involvement, advanced cardiac imaging, including echocardiography or cardiac magnetic resonance imaging, could not be performed due to the patient’s rapidly progressive clinical deterioration and critical condition. Partial clinical and biochemical improvement was observed, allowing transition to oral prednisolone at a dose of 1 mg/kg/day, with a planned gradual taper according to clinical response. Clinical deterioration occurred before tapering could be implemented. On day 20 of hospitalization, the patient developed aspiration pneumonia, followed by rapid clinical deterioration with respiratory and hepatic failure, consistent with multiorgan failure, culminating in death on day 30 of hospitalization.

## Discussion

Combination therapy with ipilimumab and nivolumab has demonstrated significant efficacy across multiple advanced malignancies but is associated with a higher incidence and severity of irAEs, particularly involving the neuromuscular and cardiovascular systems [4,10]. Among these, ICI-induced myositis is a rare but potentially life-threatening toxicity that typically occurs early after treatment initiation, often within the first 6-8 weeks, and is mediated by CD8+ T-cell-driven muscle fiber necrosis with macrophage infiltration [6,7].

This case illustrates a fulminant ICI-related neuromuscular overlap syndrome, characterized by inflammatory myositis with a suspected myasthenic component and biochemical evidence suggestive of myocardial involvement. Such overlap syndromes, involving varying combinations of myositis, myasthenia gravis, and myocarditis, are increasingly recognized and are associated with particularly severe clinical courses and high mortality rates [8-10]. Their presence reflects a broad loss of immune tolerance induced by checkpoint blockade, leading to multisystem immune-mediated injury.

ICI-induced myositis presents with a wide clinical spectrum, ranging from asymptomatic CK elevation to rapidly progressive proximal, axial, bulbar, or oculomotor weakness, which may culminate in respiratory failure [6,7]. In contrast to idiopathic inflammatory myopathies, ocular and bulbar involvement is more frequent in ICI-related disease and should prompt consideration of concomitant neuromuscular junction dysfunction. In this context, overlap with immune-mediated myasthenia gravis represents a particularly severe phenotype.

ICI-associated myasthenia gravis differs from classical idiopathic myasthenia gravis in several important aspects. It typically presents abruptly, with early bulbar and respiratory muscle involvement, frequent elevation of CK, and a markedly higher mortality rate [8]. Diagnostic criteria may be incompletely fulfilled, and many patients are seronegative. In such cases, a probable myasthenic component within an overlap syndrome may be clinically inferred based on the constellation of early ocular and bulbar symptoms, antibody positivity when present, and rapid disease progression, even in the absence of confirmatory electrophysiological testing [8,9]. This distinction is clinically relevant, as it underscores the need for urgent therapeutic escalation despite diagnostic uncertainty.

Cardiac involvement is a critical and prognostically significant component of ICI-related overlap syndromes. Myocarditis may coexist with myositis and myasthenia gravis in up to half of reported cases and is associated with mortality rates approaching 40%-60% [9,10]. In the present case, marked troponin T elevation raised strong concern for myocardial involvement. Although definitive cardiac imaging could not be performed due to the patient’s rapidly progressive clinical deterioration, the biochemical findings and clinical context were highly suggestive of immune-mediated cardiac injury. This limitation highlights the diagnostic challenges frequently encountered in fulminant presentations, where clinical instability may preclude comprehensive evaluation.

In addition to the intrinsically high mortality associated with ICI-related overlap syndromes, several patient-specific factors likely contributed to the poor outcome in this case. Advanced age may have limited physiological reserve and resilience to severe immune-mediated toxicity. Furthermore, the presence of hepatocellular carcinoma, even in the absence of overt hepatic decompensation at baseline, may have reduced hepatic reserve and impaired the capacity to tolerate systemic inflammation and infection. The history of renal cell carcinoma and active oncologic disease reflects an overall vulnerable oncologic context, potentially exacerbating susceptibility to severe irAEs. Finally, the development of aspiration pneumonia and subsequent multiorgan failure represented a critical turning point, compounding neuromuscular weakness and ultimately overwhelming recovery potential despite timely and appropriate immunosuppressive therapy.

Diagnosis of ICI-related neuromuscular toxicity relies on the integration of clinical presentation, laboratory findings, autoantibody testing, and neurophysiological studies. Electromyography typically demonstrates a myopathic pattern with active denervation, while repetitive nerve stimulation may reveal decremental responses when neuromuscular junction involvement is present. Muscle magnetic resonance imaging may show muscle edema or abnormal enhancement [12]. Muscle biopsy is generally reserved for diagnostically uncertain cases and typically reveals necrotizing myopathy with CD8+ T-cell infiltration [6]. Paraneoplastic neuromuscular syndromes remain an important differential diagnosis, and testing for onconeural antibodies may aid diagnostic clarification [13].

Although a muscle biopsy can provide definitive histopathological confirmation of ICI-induced myositis, it was not performed in this case. The patient’s fulminant clinical course, critical condition, and the need to prioritize immediate immunosuppressive therapy precluded the safe performance of an invasive diagnostic procedure. In this context, the diagnosis was supported by a compatible clinical presentation, marked elevation of muscle biomarkers, and characteristic electromyographic findings. Nevertheless, the absence of histopathological confirmation represents a limitation of this report.

Early recognition and prompt initiation of immunosuppressive therapy are essential determinants of outcome in ICI-related neuromuscular toxicity. Current guidelines recommend immediate discontinuation of ICIs and initiation of high-dose corticosteroids in moderate to severe cases, with early escalation to intravenous immunoglobulin or plasma exchange in the presence of bulbar symptoms, respiratory compromise, myocarditis, or rapid clinical progression [14,15]. Despite the timely initiation of high-dose corticosteroids and intravenous immunoglobulin in this case, only partial improvement was achieved, underscoring the aggressive nature of overlap syndromes and their frequent refractoriness to standard therapy.

This case emphasizes the importance of maintaining a high index of suspicion for neuromuscular overlap syndromes in patients receiving ICIs who develop early ocular, bulbar, or proximal muscle weakness, unexplained dyspnea, or multisystem immune-related manifestations. Even in the absence of complete diagnostic confirmation, early multidisciplinary involvement, vigilant cardiorespiratory monitoring, and rapid escalation of immunosuppressive therapy are critical, as delays are associated with poor outcomes.

## Conclusions

Overlap neuromuscular irAEs induced by ICIs, particularly the combination of ICI-associated myositis and immune-mediated myasthenia gravis, represent a fulminant clinical phenotype with high mortality, especially when bulbar or respiratory involvement is present. This case highlights the importance of early recognition of red-flag features, including acute ophthalmoparesis, bulbar dysfunction, rapidly progressive proximal weakness, marked serum CK elevation, unexplained respiratory compromise, and evidence of multisystem immune-related involvement, such as concurrent cardiac, dermatologic, or endocrine toxicities.

The coexistence of neurological manifestations with involvement of other organ systems should raise a high index of suspicion for ICI-related toxicity and prompt immediate diagnostic evaluation and therapeutic escalation. Rapid initiation of immunosuppressive therapy, vigilant cardiorespiratory monitoring, and early multidisciplinary management are essential to improve outcomes. Given the severity and high risk of recurrence associated with life-threatening overlap syndromes, permanent discontinuation of ICIs is mandatory.

## References

[1] Ribas A, Wolchok JD (2018). Cancer immunotherapy using checkpoint blockade. Science.

[2] (2024). National Cancer Institute. Common Terminology Criteria for Adverse Events (CTCAE) Version 6.0. https://ctep.cancer.gov/protocoldevelopment/electronic_applications/docs/CTCAE_v6.0.pdf.

[3] Geisler AN, Phillips GS, Barrios DM (2020). Immune checkpoint inhibitor-related dermatologic adverse events. J Am Acad Dermatol.

[4] Haugh AM, Probasco JC, Johnson DB (2020). Neurologic complications of immune checkpoint inhibitors. Expert Opin Drug Saf.

[5] Shelly S, Triplett JD, Pinto MV, Milone M, Diehn FE, Zekeridou A, Liewluck T (2020). Immune checkpoint inhibitor-associated myopathy: a clinicoseropathologically distinct myopathy. Brain Commun.

[6] Moreira A, Loquai C, Pföhler C (2019). Myositis and neuromuscular side-effects induced by immune checkpoint inhibitors. Eur J Cancer.

[7] Suzuki S, Ishikawa N, Konoeda F (2017). Nivolumab-related myasthenia gravis with myositis and myocarditis in Japan. Neurology.

[8] Safa H, Johnson DH, Trinh VA (2019). Immune checkpoint inhibitor related myasthenia gravis: single center experience and systematic review of the literature. J Immunother Cancer.

[9] Aldrich J, Pundole X, Tummala S (2021). Inflammatory myositis in cancer patients receiving immune checkpoint inhibitors. Arthritis Rheumatol.

[10] Pathak R, Katel A, Massarelli E, Villaflor VM, Sun V, Salgia R (2021). Immune checkpoint inhibitor-induced myocarditis with myositis/myasthenia gravis overlap syndrome: a systematic review of cases. Oncologist.

[11] Oken MM, Creech RH, Tormey DC, Horton J, Davis TE, McFadden ET, Carbone PP (1982). Toxicity and response criteria of the Eastern Cooperative Oncology Group. Am J Clin Oncol.

[12] Daoussis D, Kraniotis P, Filippopoulou A (2020). An MRI study of immune checkpoint inhibitor-induced musculoskeletal manifestations myofasciitis is the prominent imaging finding. Rheumatology (Oxford).

[13] Graus F, Delattre JY, Antoine JC (2004). Recommended diagnostic criteria for paraneoplastic neurological syndromes. J Neurol Neurosurg Psychiatry.

[14] Schneider BJ, Naidoo J, Santomasso BD (2021). Management of immune-related adverse events in patients treated with immune checkpoint inhibitor therapy: ASCO guideline update. J Clin Oncol.

[15] Brahmer JR, Lacchetti C, Schneider BJ (2018). Management of immune-related adverse events in patients treated with immune checkpoint inhibitor therapy: American Society of Clinical Oncology clinical practice guideline. J Clin Oncol.

